# Association between chronic kidney disease stages and changes in ambulatory blood pressure monitoring parameters

**DOI:** 10.1590/2175-8239-JBN-2024-0128rpen

**Published:** 2024-11-01

**Authors:** André Murad Nagahama, Vanessa dos Santos Silva, Vanessa Burgugi Banin, Roberto Jorge da Silva Franco, Pasqual Barretti, Silmeia Garcia Zanati Bazan, Luis Cuadrado Martin

**Affiliations:** 1 Universidade Estadual Paulista, Faculdade de Medicina, Botucatu, SP, Brazil.

We are grateful to Drs. Daungsupawong and Wiwanitkit for dedicating their time to read and provide comments on our article^1,2^. Initially, you emphasize that this was a cross-sectional study and, therefore, it is not possible to establish causal relationships. We agree with this observation, and were careful to avoid mentioning any relationships in the article^2^.

As for the single-center nature of the study, we acknowledge this aspect and have addressed it in the “discussion” section, specifically in the part focused on the study’s limitations.

Regarding confounding factors, the associations were adjusted for age, gender, body mass index, smoking, cause of CKD and number of antihypertensive drug classes used. These are the main factors contributing to potential confounding. Thus, we can confirm that the observed associations remain consistent even when accounting for the variables evaluated. As for the other limitations mentioned, it is not possible to provide further comments since they are indeed a limitation of the study.

In terms of the accuracy of CKD staging, we mentioned in the “limitations” section that we lacked complete data on albuminuria; therefore, we were unable to stage it according to KDIGO classification category “A”.

You comment on the fact that our work does not address changes in medications over the course of the study nor does it examine the effects of CKD treatment. Our study^2^, as previously mentioned, is a cross-sectional study, which limits our ability to assess continuity and changes in therapeutics over time. Furthermore, as a cross-sectional study, it does not allow for a comparison of therapies aimed at achieving better outcomes. This would only be possible in a double-blind randomized clinical trial study.

It is essential and highly relevant that future studies, using longitudinal designs and clinical trials, be conducted to better understand the effect of chronic kidney disease on ABPM parameters.

Finally, we would like to emphasize that our study^2^ is a single-center cross-sectional study which benefited from a large sample size. By conducting a generalized linear regression statistical analysis, we could demonstrate associations while accounting for the main confounding factors when analyzing ABPM parameters across different stages of CKD, a finding that is not frequently reported in the literature.

## References

[1] Daungsupawong H, Wiwanitkit V (2024). Association between chronic kidney disease stages and changes in ambulatory blood pressure monitoring parameters.. J Bras Nefrol..

[2] Nagahama AM, Silva VDS, Banin VB, Franco RJDS, Barretti P, Bazan SGZ (2024). Association between chronic kidney disease stages and changes in ambulatory blood pressure monitoring parameters.. J Bras Nefrol..

